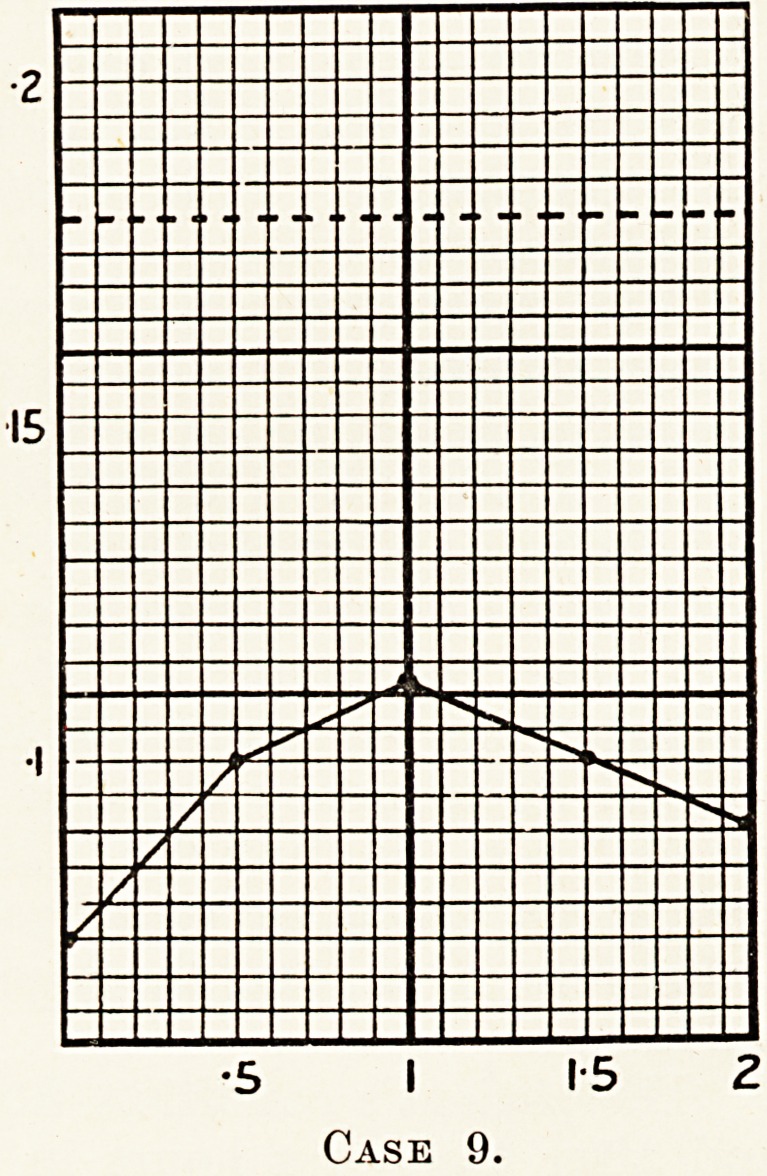# Sugar Tolerance Tests
*A Martyn Memorial Scholarship Essay.


**Published:** 1928

**Authors:** Hugh D. Pyke


### SUGAR TOLERANCE TESTS.*

BY

HUGH D. PYKE.

By way of illustrating the value of these tests, a series of cases is submitted, in each of which a glucose or lævulose tolerance test has been performed.

The method used has been that practised by Hugh Maclean.[1]

All the patients on whom these tests have been performed have been in- or out-patients of the Bristol General Hospital. I am indebted to the physicians under whose care they were for permission to publish these records, and to Professor Hadfield for advice and help.

To obtain a graph for comparative purposes, a test on a normal person (H. D. P.) was carried out. The first reading of the percentage blood sugar represents that of a specimen of blood taken four hours and a half after breakfast. Fifty grams of glucose were then taken by mouth, and readings taken at half-hourly intervals during the first two hours after taking the glucose. The specimens of blood were taken at times similar to these in all the cases cited.

The readings obtained were as follows: 0·093, 0·125, 0·162, 0·112, 0·081. These readings may be expressed graphically. In this and all the following graphs the vertical represents the percentage blood sugar, the horizontal the time in hours and the dotted horizontal the normal renal threshold.

**Case 1.**—Female, aged 30. She had twins in October, 1925. In the following January her menstrual periods restarted,

* A Martyn Memorial Scholarship Essay.

accompanied by pains in the back. She was treated for dyspepsia. In March, 1926, the patient found she was losing weight rather rapidly. Sugar was discovered in the urine, which disappeared after one week's dieting. Intermittent attacks of glycosuria occurred until May, 1926, when the urine was found to be loaded with sugar, and xanthelasma had developed around the inner canthus of each eye.

At a glucose tolerance test performed on May 27th, 1927, the readings obtained were: 0·231, 0·330, 0·387, 0·428, 0·387.

A specimen of urine taken after the experiment revealed the presence of a heavy percentage of sugar.

This, then, was the case of a young woman of healthy appearance who, except for the loss of weight and the xanthelasma, was practically without diabetic symptoms; yet the glucose tolerance test proved her to be a true diabetic. This demonstrates the necessity of applying the test to all such cases of glycosuria.

**Case 2.**—Male, aged 21. This patient found that he developed glycosuria after taking any considerable quantity of sweet food. Otherwise he had absolutely no symptoms, and was anxious as to whether he was an early diabetic or not.

The glucose tolerance test done on July 5th, 1927, showed that his case was one of a typical alimentary glycosuria.

The readings of his case were as follows: 0·093, 0·150, 0·206, 0·106, 0·075. Sugar appeared in the urine one hour after taking the glucose.

**Case 3.**—A young woman admitted into hospital May 14th, 1927, with severe abdominal pain, localised in the right iliac fossa. While she was in hospital sugar was found in her urine. The blood sugar, estimated four hours after food, was 0·143. She was discharged from hospital (urine sugar-free) on a diabetic diet. She attended out-patients regularly, but the

CASE 1.

CASE 2.

glycosuria never reappeared on the diet (1,760 calories). No other symptoms of diabetes developed.

It was discovered that the patient had been suckling her child for the six months preceding her admission into hospital in May. Was it actually glucose or lactose that had been present in the urine ?

A glucose tolerance test done on June 22nd, 1927, gave the following result: 0·093, 0·112, 0·150, 0·118, 0·081. No glucose or lactose was found in the urine after the tolerance test by the phenyl-hydrazine method.

The diagnosis, therefore, was one of lactosuria.

**Case 4.**—Male, aged 42. Sixteen months before admission he had fallen unconscious after sneezing. This was followed by months of giddiness, buzzing noises in ears, vomiting and headaches. No aural or ocular lesion was found. The blood gave a triple positive Wassermann reaction. There were transient attacks of glycosuria.

A glucose tolerance test done in July, 1927, gave the following readings: 0·106, 0·193, 0·137, 0·068, 0·056. A specimen of urine taken before the test was sugar-free, another obtained an hour after taking the glucose contained 3·3 per cent. sugar, while a third specimen obtained at the end of the test contained 1·2 per cent. sugar.

CASE 3.

CASE 4.

The test, therefore, revealed a decreased sugar tolerance on the part of the patient. It was concluded that the glycosuria was due to a lesion of the pituitary gland, and was connected with his other symptoms. The presence of a cranial tumour was diagnosed.

In August of the same year—one month after the test—the patient was readmitted into hospital with papillœdema and other signs of increased intracranial pressure. Repeated lumbar punctures gave the patient marked relief, but he soon developed Cheyne-Stokes breathing and died. At the autopsy a large cyst of the cerebellum was found replacing the middle lobes and obstructing the iter. A polycystic condition of the kidneys was also found with cyst-adenomatosis of the pancreas. Death was due to acute hydrocephalus.

**Case 5.**—Male, aged 14. Admitted for severe jaundice. He worked in a large oil and petrol works. In January, 1927, he gradually became jaundiced. This persisted until June, 1927. when he was admitted to hospital. The stools had been a little loose and putty-coloured, and he complained of lassitude, but otherwise he felt quite well. On examination the spleen was found to be palpable and the liver enlarged and hard. No ascites. Urine dark in colour. A Van den Bergh test done on June 26th, 1927, showed a delayed and weakly positive direct

CASE 5.

reaction, and a strongly positive indirect reaction. In view of a possible diagnosis of acholuric jaundice, the fragility of the blood cells was tested and found to be normal.

A lævulose test for hepatic inefficiency (50 grams used) was markedly positive. The readings were as follows: 0·093, 0·256, 0·181, 0·075, 0·062.

As benzol and allied substances are well known as hepatic poisons producing all grades of liver necrosis, the diagnosis was arrived at of subacute hepatic necrosis, due probably to the inhalation of benzol fumes.

**Case 6.**—Male, aged 18. He was admitted for excessive polyuria, 24 pints of urine were being passed in as many hours. He complained of thirst and lassitude, otherwise he felt fit. He had been discharged from army because of this complaint. The Wassermann reaction (blood) was negative. The urine had a specific gravity of 1005, and contained no sugar or albumin. No acidosis was present.

A diagnosis of diabetes insipidus was made, and a glucose tolerance test performed to confirm the diagnosis. The readings were as follows: 0·056, 0·062, 0·056, 0·050, 0·050. This result showed the patient to possess an increased sugar

CASE 6.

CASE 7.

tolerance, probably of pituitary origin, an observation which supports the view that the pituitary gland is the organ at fault in diabetes insipidus.

**Case 7.**—Male, aged 35. He was admitted into hospital complaining of difficulty in walking and controlling the voluntary movements of the limbs. The onset followed an attack of chorea at twelve years of age. His gait was of the spastic scissor-legged type. He had a coarse tremor of the head and limbs that was increased in all voluntary movements. Intellect and memory good. Poor articulation. Some nystagmus. No jaundice. Liver not palpable. The Wassermann reaction (blood) was negative. The possibility of progressive lenticular degeneration was considered, and a lævulose test for hepatic inefficiency was done on August 21st, 1927, and the following readings obtained: 0·081, 0·086, 0·081, no specimen, 0·086. The test showed that the cirrhosis of the liver—if present at all—was not severe enough to destroy the efficiency of the hepatic cells. But as the cirrhosis produces no obvious symptoms in this disease, it is only in keeping with the diagnosis that the liver should show no inefficiency sufficiently severe to give a positive test.

**Case 8.**—Woman, aged 50. She had buzzing noise in ears during the ten years previous to admission into hospital, accompanied by a vague history of dyspepsia, nausea, thirst

CASE 8.

CASE 9.

and headache. Her liver was enlarged and palpable, well below costal margin. No jaundice. A diagnosis of subacute hepatitis.

In a lævulose tolerance test done February 9th, 1927, the readings showed no marked hepatic inefficiency, *i.e.* 0·093, 0·112, 0·093, 0·106, 0·112.

**Case 9.**—Male, aged 45. Nausea, epigastric pain and flatulence of many years' duration. Barium meal showed no organic lesion of alimentary canal. No jaundice. Liver enlarged as in preceding case.

A lævulose test performed on this patient gave a very similar result to that of the preceding case, the readings being: 0·075, 0·100, 0·112, 0·100, 0·093.

The distinction between the last two cases cited (chronic hepatitis) and Case 5 (subacute hepatic necrosis) is well stressed by the results of the lævulose tests. In the case of subacute hepatic necrosis the lesion is severe, very diffuse, and involves the parenchyma, as the toxic benzol compounds are probably conveyed by the blood stream. In the other two cases the lesion is less diffuse, and mainly affects the connective tissue. The parenchyma is thus left sufficiently healthy to give a negative result with the hepatic inefficiency tests.

**Case 10.**—The next case is not one in which a full sugar tolerance test was done, as the diagnosis was unfortunately only too obvious. It illustrates, however, the type of case of severe diabetes which fails to respond to insulin treatment.

The patient was a married woman, aged 40. She was admitted into hospital on July 6th, 1927, in a comatose condition. She had a history of two years' diabetes, but had never received insulin treatment. The breath smelt sweet, she had air hunger, and the urine showed a heavy percentage of sugar and acidosis. Forty units of insulin were given, and at 5 p.m. on July 6th, 1927, the percentage of blood sugar proved to be 0·478. Repeated forty-unit doses of insulin were given, but the patient showed no tendency to come out of her coma.

At 7 p.m. on July 6th, 1927, after 220 units of insulin had been given, the blood sugar was still up to 0·443. Another 80 units were given, and the blood sugar percentage fell to 0·400 only.

The patient's condition, however, showed no improvement, and after thirty-six hours in hospital she died, although 300 units of insulin had been given in that time, with very little fall in the percentage of sugar in the blood.

### SUMMARY.

A series of cases is described to exemplify the diagnostic value of estimations of the body's tolerance of glucose and lævulose, in differentiating the various kinds of glycosuria and investigating the efficiency of the liver.

### REFERENCE.

[1] Hugh Maclean, M.D., *Modern Methods in the Diagnosis and Treatment of Glycosuria and Diabetes*, 2nd Edition. 1924.

## Figures and Tables

**Figure f1:**
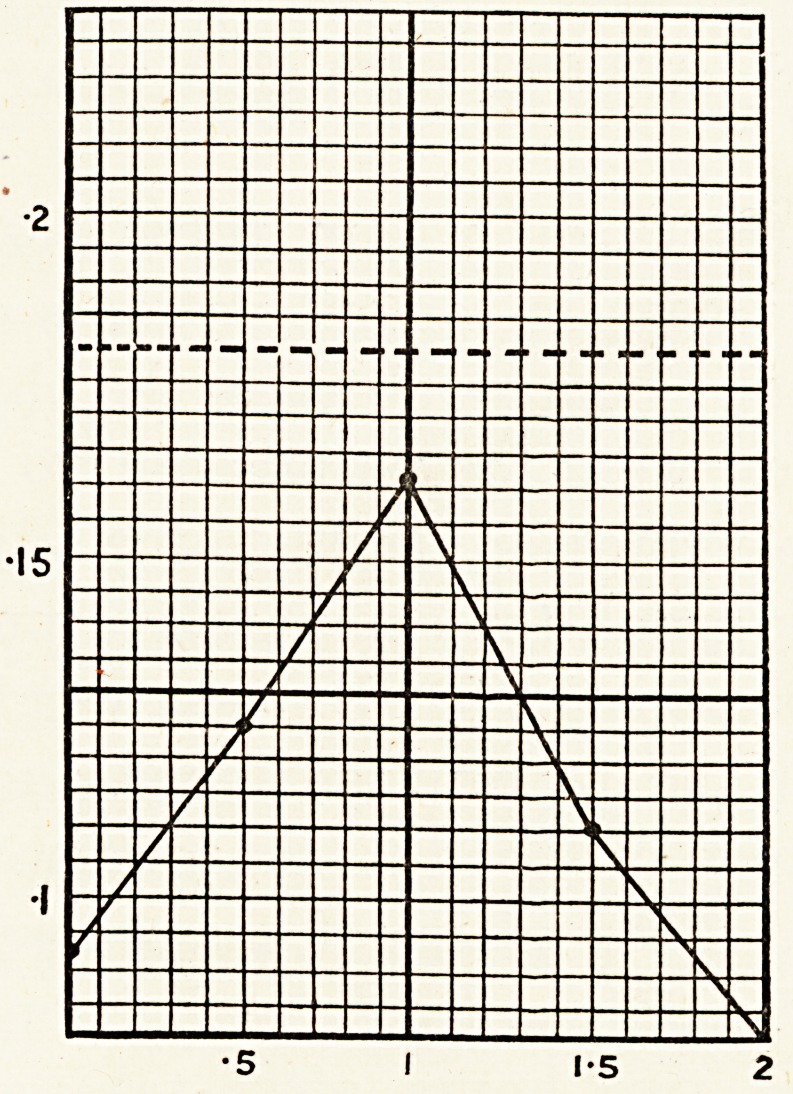


**Case 1. f2:**
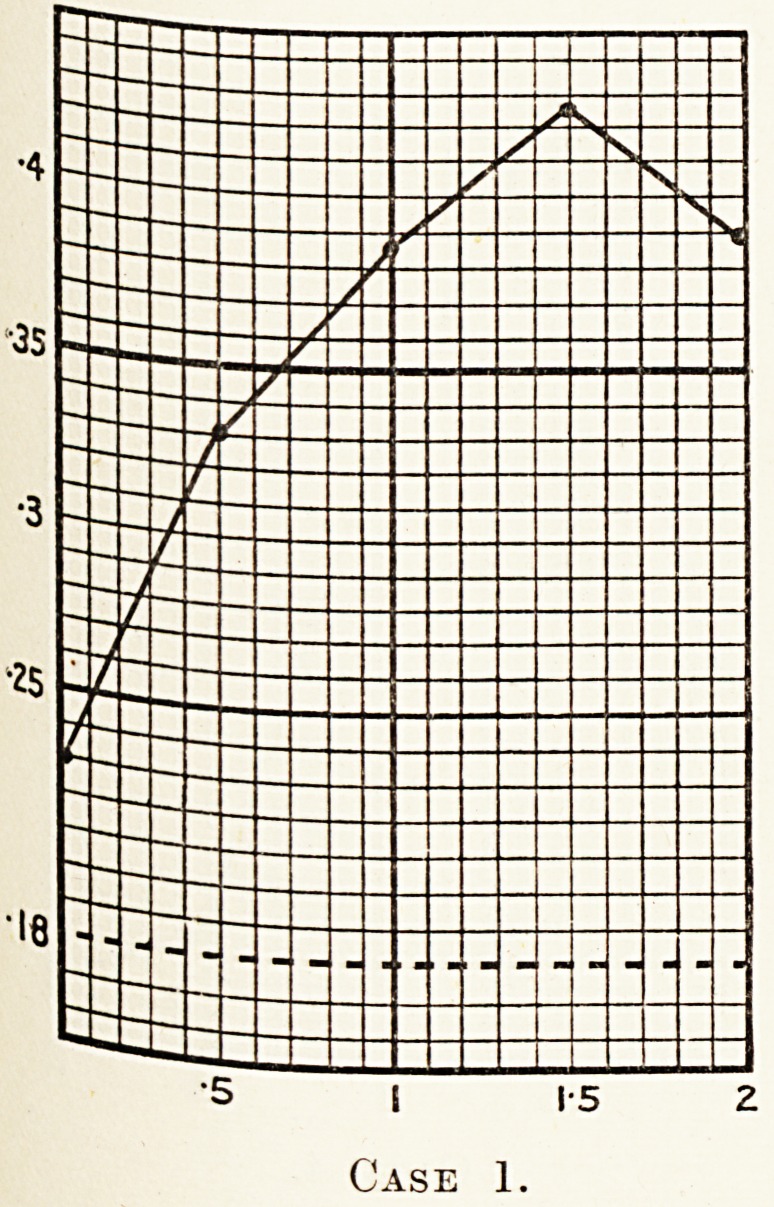


**Case 2. f3:**
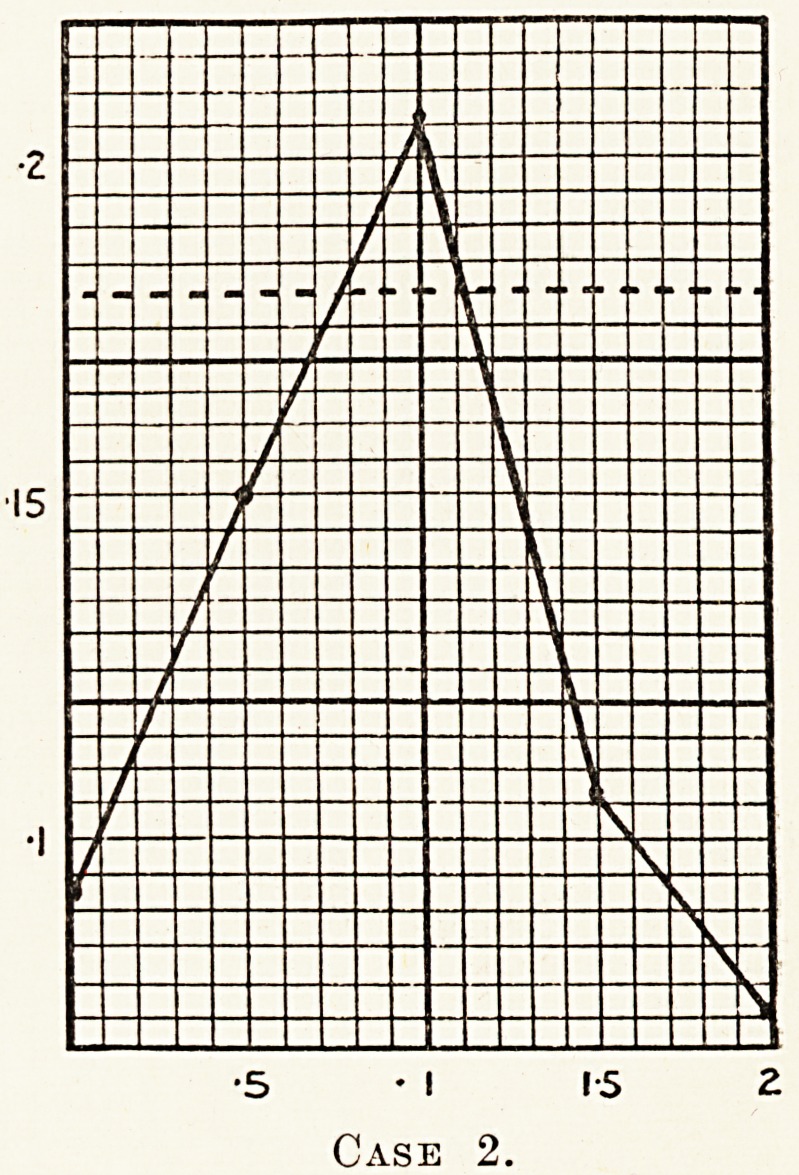


**Case 3. f4:**
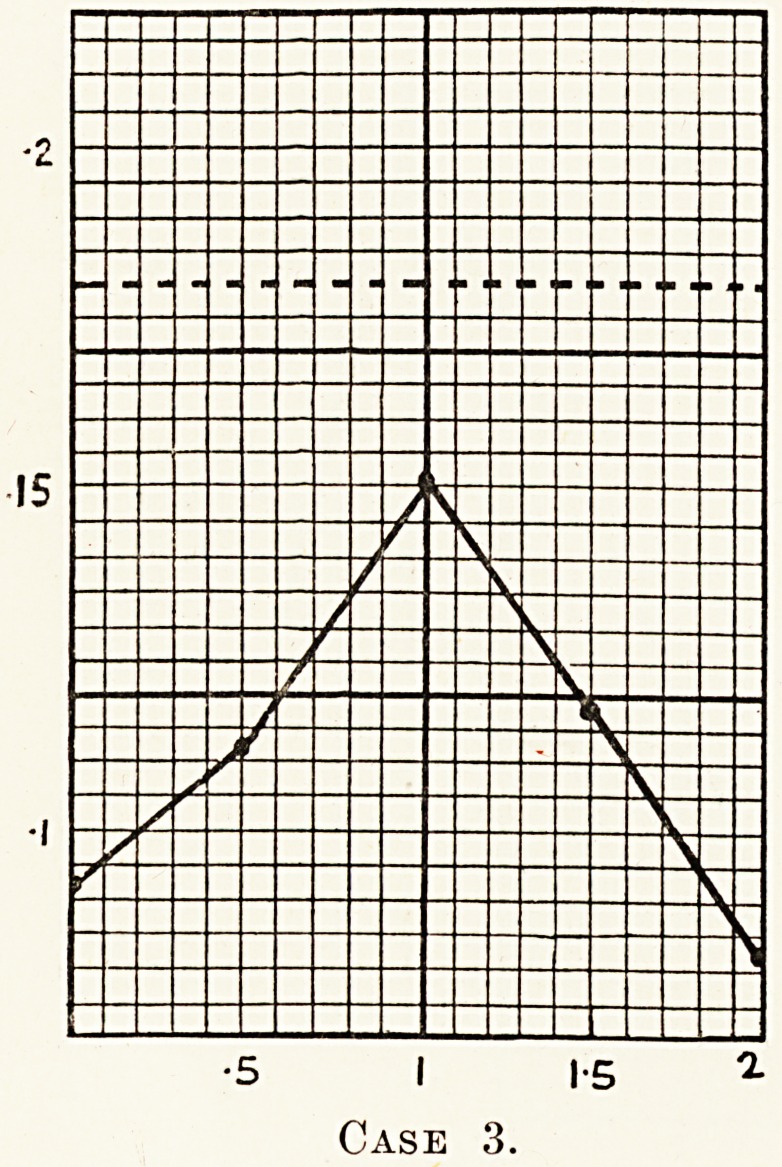


**Case 4. f5:**
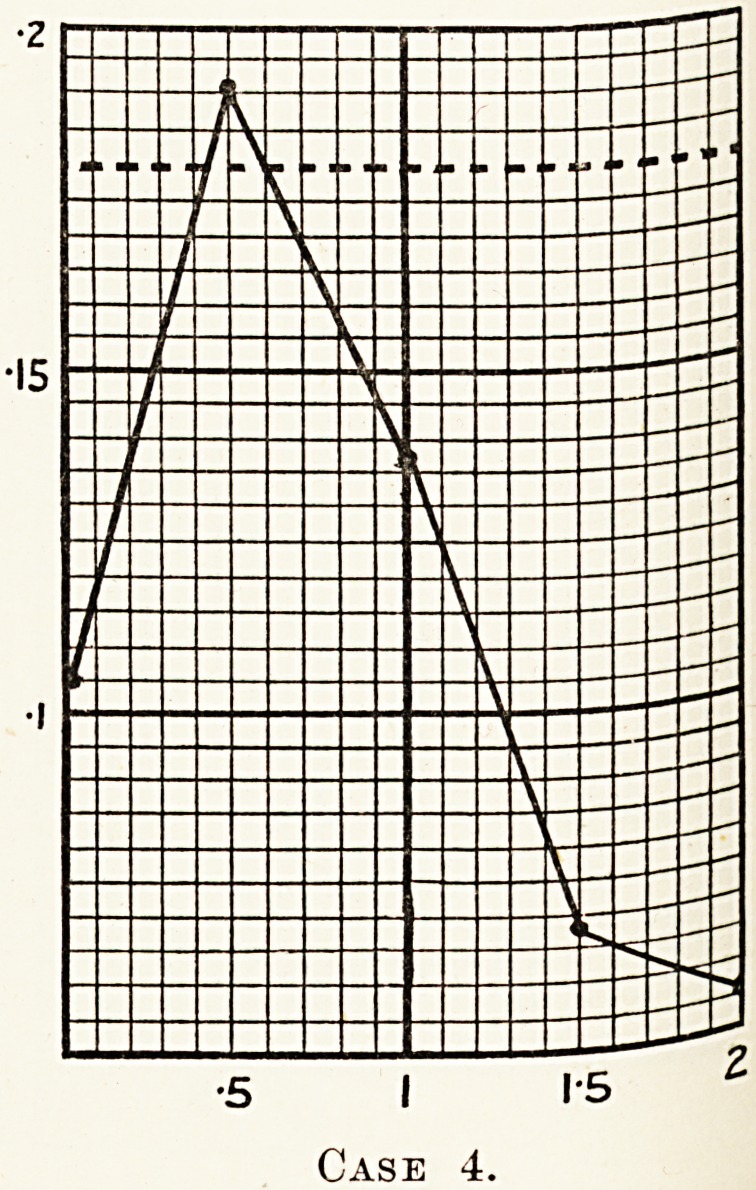


**Case 5. f6:**
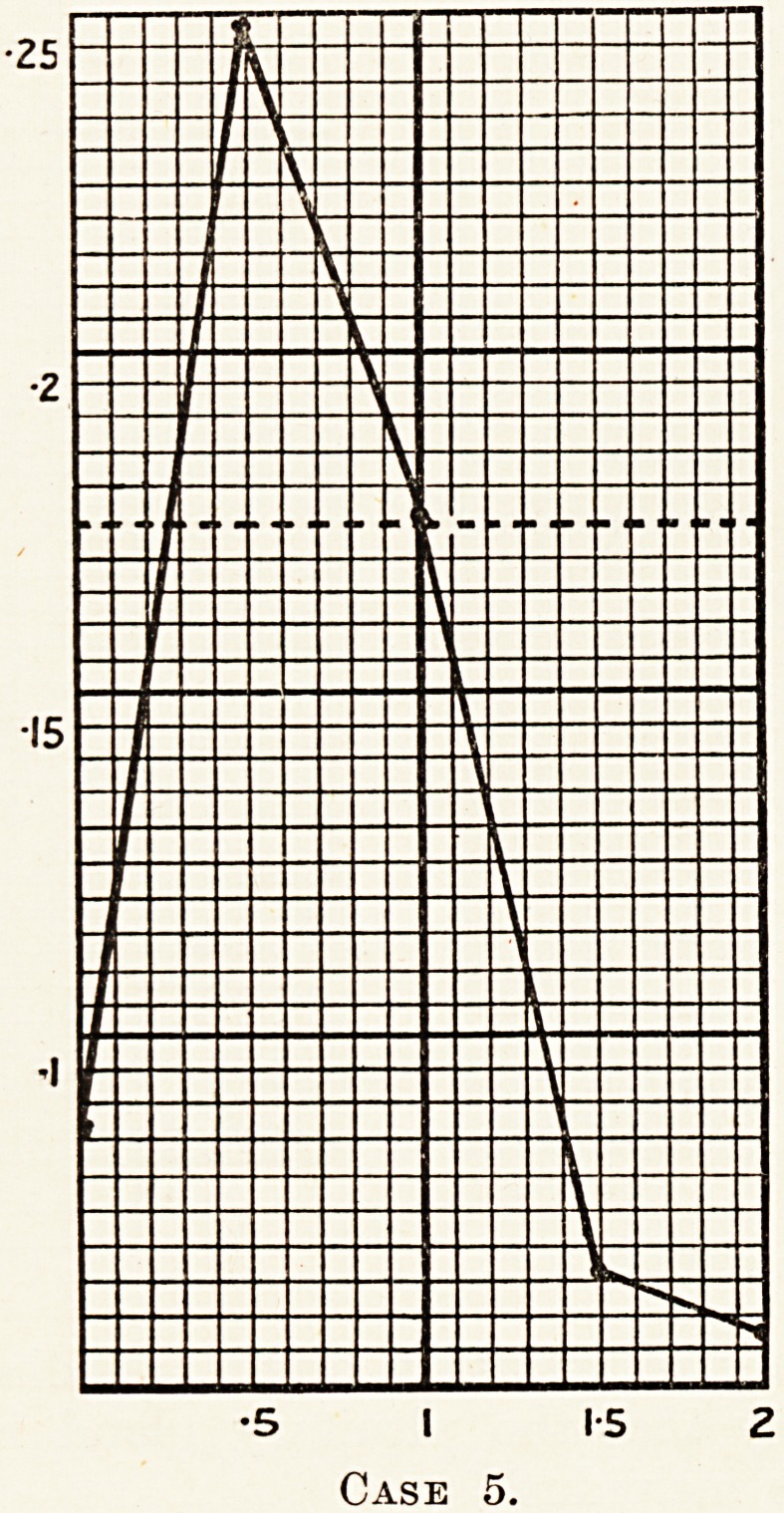


**Case 6. f7:**
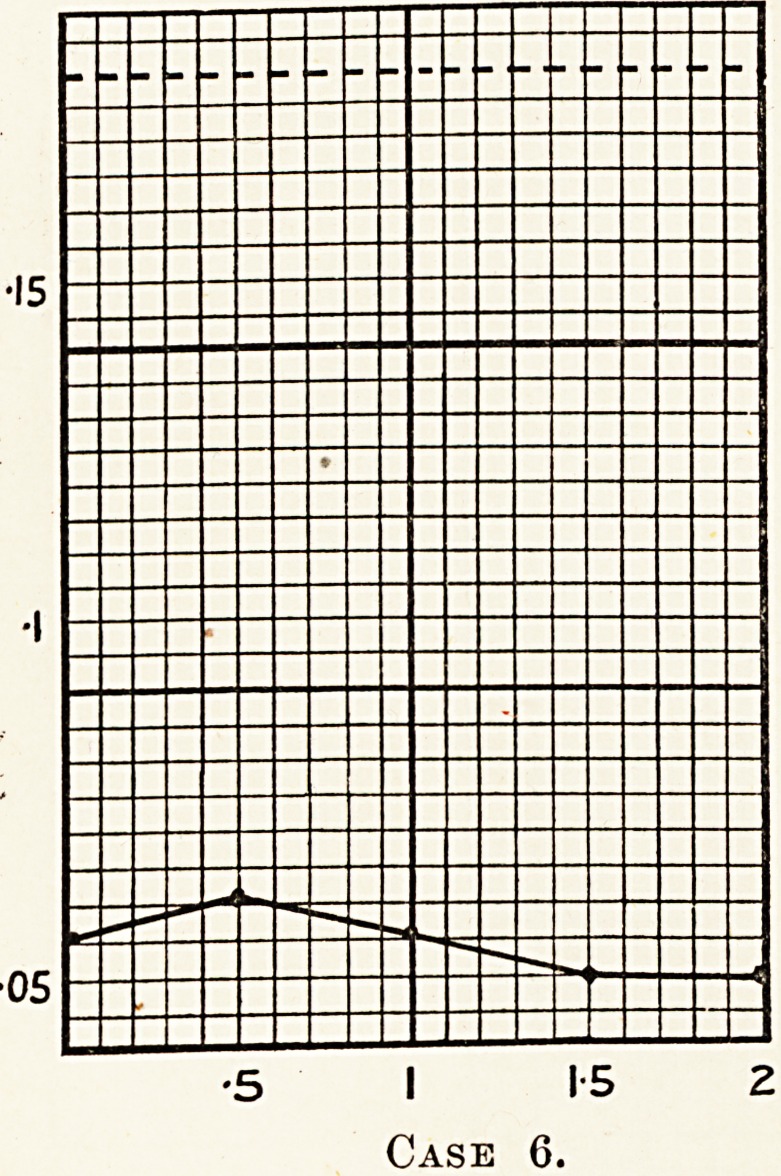


**Case 7. f8:**
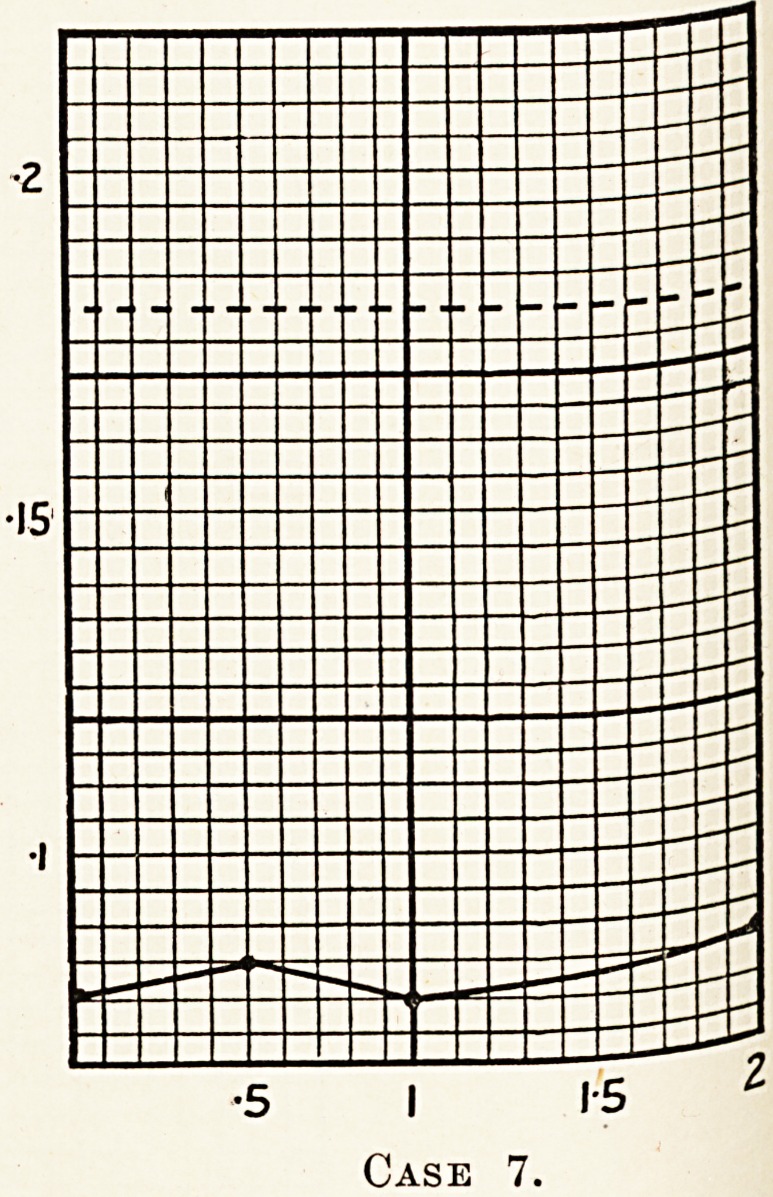


**Case 8. f9:**
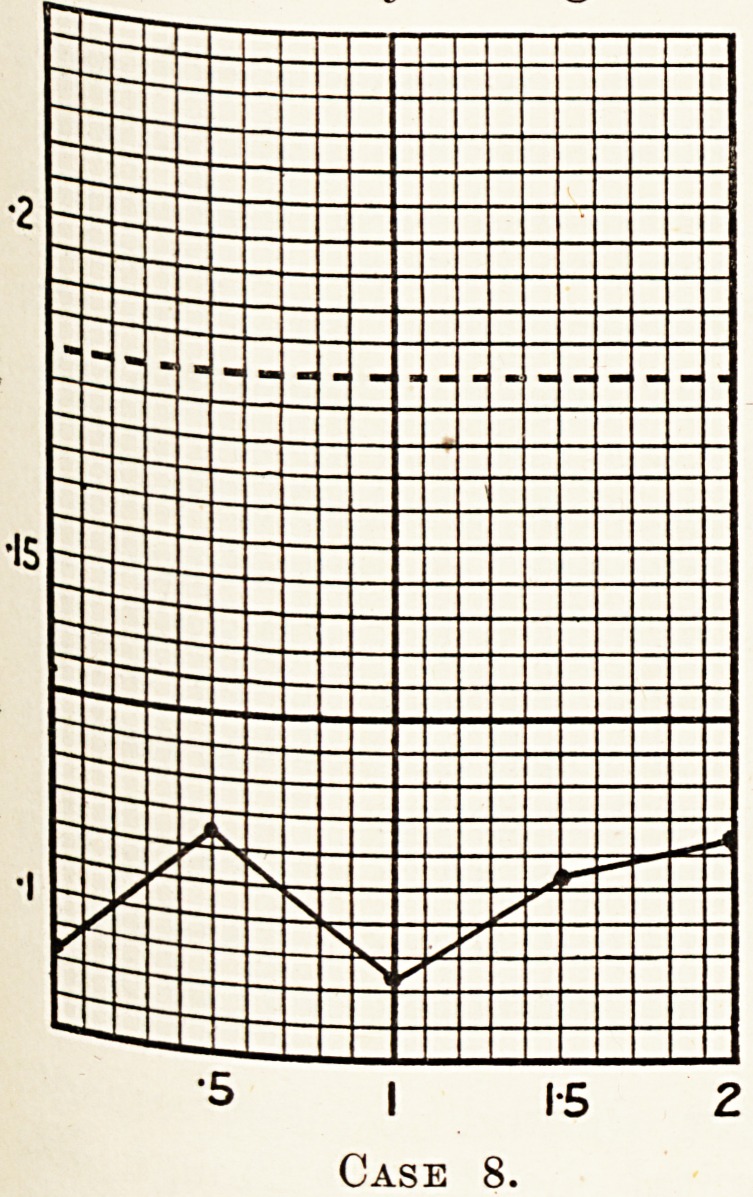


**Case 9. f10:**